# Insulin-degrading enzyme regulates insulin-directed cellular autoimmunity in murine type 1 diabetes

**DOI:** 10.3389/fimmu.2024.1474453

**Published:** 2024-11-12

**Authors:** Marie-Andrée Bessard, Anna Moser, Emmanuelle Waeckel-Énée, Vivian Lindo, Abdelaziz Gdoura, Sylvaine You, F. Susan Wong, Fiona Greer, Peter van Endert

**Affiliations:** ^1^ Université Paris Cité, Institut National de la Santé et Recherche Médicale (INSERM), Centre National de La Recherche Scientifique (CNRS), Institut Necker Enfants Malades, Paris, France; ^2^ M-SCAN, Wokingham, United Kingdom; ^3^ Université Paris Cité, Institut National de la Santé et Recherche Médicale (INSERM), Centre National de La Recherche Scientifique (CNRS), Institut Cochin, Paris, France; ^4^ Institute of Molecular and Experimental Medicine, School of Medicine, Cardiff University, Cardiff, United Kingdom; ^5^ Service Immunologie Biologique, Assistance Publique - Hôpitaux de Paris (AP-HP), Hôpital Universitaire Necker-Enfants Malades, Paris, France

**Keywords:** type 1 diabetes, insulin, antigen presentation, antigen processing, non-obese diabetic mouse, CD8^+^ T cell

## Abstract

Type 1 diabetes results from the destruction of pancreatic beta cells by autoreactive T cells. As an autoantigen with extremely high expression in beta cells, insulin triggers and sustains the autoimmune CD4^+^ and CD8^+^ T cell responses and islet inflammation. We have previously shown that deficiency for insulin-degrading enzyme (IDE), a ubiquitous cytosolic protease with very high affinity for insulin, induces endoplasmic reticulum (ER) stress and proliferation in islet cells and protects non-obese diabetic mice (NOD) from diabetes. Here we wondered whether IDE deficiency affects autoreactive CD8^+^ T cell responses to insulin and thereby immune pathogenesis in NOD mice. We find that *Ide^-/-^* NOD harbor fewer diabetogenic T cells and reduced numbers of CD8^+^ T cells recognizing the dominant autoantigen insulin and islet-specific glucose-6-phosphatase catalytic subunit-related protein (IGRP). Using *in vitro* digestions and cellular antigen presentation assays, we show that generation of the dominant insulin epitope B_15-23_ involves both the proteasome and IDE. IDE deficiency attenuates MHC-I presentation of the immunodominant insulin epitope by beta cells to cognate CD8^+^ T cells. Consequently, *Ide^-/-^* islets display reduced susceptibility to autoimmune destruction upon grafting, and to killing by insulin-specific CD8^+^ T cells. Moreover, *Ide^-/-^* mice are partly resistant to disease transfer by CD8^+^ T cells specific for insulin but not for IGRP. Thus, IDE has a dual role in beta cells, regulating ER stress and proliferation while at the same time promoting insulin-directed autoreactive CD8^+^ T cell responses.

## 1 Introduction

Type 1 diabetes (T1D) results from the failure of immune regulation to control autoreactive T lymphocytes that recognize and ultimately destroy pancreatic beta cells in a chronic inflammatory process. Both CD8^+^ cytotoxic and CD4^+^ helper T cells recognize beta cell antigens and play critical roles in the pathogenesis of T1D (1). For example, CD4^+^ T cell clones can transfer diabetes (2); on the other hand, non-obese diabetic (NOD) mice lacking MHC class I molecules develop neither insulitis nor diabetes (3). Uptake of dying beta cells by islet-infiltrating professional antigen presenting cells (APCs) results in (cross-) presentation of islet antigens to CD4^+^ and CD8^+^ T cells which are primed in pancreatic lymph nodes or in islets and can differentiate into effector T cells killing beta cells directly and/or through secretion of inflammatory cytokines (4).

Among beta cell antigens, (pro-)insulin (PI) plays a particularly prominent role (5). In the model of the non-obese diabetic (NOD) mouse, diabetes and islet inflammation are both prevented in mice expressing insulin molecules unable to stimulate immunodominant CD4^+^ and CD8^+^ T cells due to a single amino acid substitution in overlapping immunodominant T cell epitopes (6), suggesting that the autoimmune process is triggered by T cell recognition of insulin. Insulin-specific CD8^+^ T cells emerge earlier than cells recognizing other dominant beta cell antigens in the NOD mouse, are highly prevalent in islets from T1D patients, and can destroy human and murine beta cells (5). Moreover, the level of insulin presentation during thymic T cell education affects diabetes rate and onset in the NOD model (7).

Considering the critical role of insulin in the pathogenesis of T1D, it is of interest to study the intracellular pathways involved in the presentation of derived peptides by MHC molecules that are insufficiently understood. Previous studies have characterized the non-canonical processing pathway for preproinsulin signal peptide-derived HLA class I-restricted epitopes; these peptides require cleavage by the intramembrane enzyme signal peptide peptidase, ejection into the cytoplasm, active import into the endoplasmic reticulum (ER) and trimming by ER peptidases (8). Conversely, generation of insulin epitopes recognized by CD4^+^ T cells by lysosomal proteases can involve transpeptidation events creating hybrid peptides containing different fragments of insulin or of insulin with other beta cell proteins (9). Moreover, the isolated insulin B chain is efficiently degraded to numerous fragments by the cytosolic proteasome, suggesting its involvement in presentation for MHC-I restricted presentation (10). However, the role of another protease with ubiquitous expression and very high affinity for insulin, namely insulin-degrading enzyme (IDE; also called insulinase) in insulin antigen processing and presentation has never been studied. IDE is mainly localized in the cytosol and thus is unlikely to have access to proinsulin in the secretory pathway or secretory granules. However, especially in situations of metabolic or proteotoxic stress, proinsulin can be ejected from the ER to the cytosol through the ER-associated degradation (ERAD) pathway (11), thus becoming a potential substrate for processing by cytosolic proteases to epitopes presented by MHC-I molecules on beta cells (12).

The role of IDE in insulin metabolism is poorly understood. IDE-deficient hepatocytes lose 80% of their insulin-degrading capacity (13) suggesting a role for IDE in removal of circulating insulin, although another study reported degradation of internalized insulin by cathepsin D (14). *Ide^-/-^* mice display hyperinsulinemia according to some authors but not to others (15–18). *Ide^-/-^* mice are glucose intolerant (15, 16, 18, 19). Additional evidence suggesting a role of IDE as “dead-end chaperone” (20, 21) and in regulation of proteasome activity and autophagy (22, 23) is consistent with complex and as yet poorly understood functions of IDE (24).

We have recently found that IDE deficiency induces low-level ER stress both in pancreatic murine and human beta cells and in hepatocytes (25, 26). In beta cells, this induces proliferation enhanced by proteotoxic stress. Moreover, *Ide* ^-/-^ NOD islets upregulate the beta cell-protective protein Reg2, and *Ide^-/-^* NOD mice display strongly reduced diabetes incidence, presumably resulting at least in part from enhanced proliferation and Reg2-induced regeneration (25). However, it remained unclear whether IDE played a role in processing and presentation to CD8^+^ T cells of the key autoantigen insulin. Here we analyzed processing and T cell recognition of the strongly immunodominant epitope insulin B_15-23_, combining *in vitro* digestions, examination of T cell responses *ex vivo* and adoptive transfer of pathogenic CD8^+^ T cells. We report selective alteration of autoreactive insulin-specific T cell responses in *Ide^-/-^* NOD mice. Thus, IDE has a dual role in beta cells, regulating ER stress and proliferation while at the same time promoting insulin-directed autoreactive CD8^+^ T cell responses.

## 2 Materials and methods

### 2.1 Mice

IDE-deficient C57BL/6 mice have been described previously (16). These mice had been derived at Taconic Lexicon Genetics (The Woodlands, TX) from ES cells with a mixed 129SvEvBrd x C57BL/6 background and subsequently backcrossed more than 10 generations to the C57BL/6 strain. IDE-deficient NOD mice were produced by back-crossing this strain 12 times to standard NOD mice bred in the animal facility of INSERM U1151, initially using a speed-backcross approach (27). This was done using a panel of 36 informative microsatellite markers covering the *Idd1* to *Idd20* susceptibility loci of the NOD mouse. All allelic loci of the NOD genotype were fixed at generation 5. *Ide^+/+^* NOD mice were bred in house and WT C57/BL6 mice purchased from Janvier (Saint-Berthevin, France). Animal experimentation performed in this study was approved by the Comité Régional d’Éthique pour l’Expérimentation Animale Ile de France – René Descartes (n deg; P2.LS.012.06).

### 2.2 ELISPOT assays

Blood was collected from anesthetized mice by cardiac puncture using a heparinized syringe and diluted in an equal volume of RPMI 1640 medium with 10% FCS (Invitrogen). Cells were pelleted by centrifugation at 500 x *g* for 10 min at room temperature, followed by lysis of red blood cells by incubation in 5 ml of 150 mM NH_4_Cl, 1 mM KHCO_3_, and 0.1 mM EDTA for 5 min. Finally, white blood cells were pelleted, resuspended in complete RPMI 1640 medium, and used for ELISPOT assays. Lymphocytes from spleens were obtained by forcing organs through a 40 µm cell strainer, followed by filtering, washing in HBSS, red blood cells lysis and resuspension in complete RPMI 1640 medium. Ninety-six-well polyvinylidene difluoride plates (Millipore) were coated overnight with an anti-IFN-γ Ab (U-CyTech Biosciences Utrecht, The Netherlands). Plates were blocked for 1 hr at 37°C with 200 µl/well of RPMI 1640 medium with 10% FCS. Lymphocytes were then added in a total volume of 200 µl/well of RPMI 1640 medium supplemented with 10% FCS and 1 U/ml IL-2. 2x 10^5^/well irradiated (3500 rad) splenocytes were used as APCs in ELISPOT assays examining peripheral blood lymphocytes which were added at 2 x 10^4^ cells/well. CD3 mAb 145 2C11 was used as positive control at a concentration of 1 µg/ml. Peptides were added at 7 µM and incubated with cells at 37°C for 24 h. Then the plates were washed, and a biotinylated anti-murine IFN-γ mAb (U-CyTech Biosciences) diluted in PBS with 10% FCS was added. Following incubation for 2 hrs at room Temperature, alkaline phosphatase-conjugated ExtrAvidin (Sigma-Aldrich) diluted in PBS with 0.5% FCS was added for a 1 hr incubation at room temperature. Spots were visualized by addition of bromo-4-chloro-3-indolyl phosphate/nitro blue tetrazolium substrate (Sigma-Aldrich), air drying and counting using an AID reader (Autoimmun Diagnostika). All data are means of sextuplicate wells and expressed as spot-forming cells (SFC) per 10^6^ responder cells. A receiver-operator characteristics analysis was performed to define the cut-off for positive responses. The cut-off was set at the average basal reactivity + 3 SD, as this was the value giving the best sensitivity and specificity.

### 2.3 Quantification of islet-infiltrating lymphocytes and APCs

To identify islet-infiltrating lymphocytes, hand-picked islets from 9- and 20-week-old mice were dissociated using cell dissociation buffer and stained with two panels of mAbs with the following specificities: CD4-Pacific Blue, Foxp3-APC, CD69-FITC and CD25-PE for CD4^+^ T cells, and CD8-Pacific Blue, CD62L-APC, CD69-FITC and CD44-PE for CD8^+^ T cells (all Abs from BD Biosciences). Samples were acquired on a BD FACSCanto II cytometer (BD Biosciences) and analyzed using FlowJo v10 software.

Islet-infiltrating APCs were quantified independently using our standard method, i.e. hand-picking islets under a binocular following dissociation to single cells, and using a density gradient as described below. In the latter method, collagenase P-liberated islets were first sedimented at 146 x *g* for 2min. The islet pellet was overlaid with solutions containing (bottom to top) 30, 23, 20 and 11% of Ficoll diluted in HBSS, and the gradient was centrifuged at 1037 x g for 17min at RT without break. Islets were recovered at the 11/20 and 20/23 interfaces, washed first in HBSS and then in PBS, dissociated in enzyme-free cell dissociation buffer and finally resuspended in HBSS with 5% FCS and 1% Hepes. Islet-infiltrating lymphocytes were then identified and characterized by staining with mAbs with the specificities and fluorochrome conjugations as follows: CD19-PE/AF594 (clone 6D5), CD11c-BV421 (clone N418), CD86-BV-510 (clone GL-1), CD11b-PE-Cy7 (clone M1/70), CD80-PE (clone 16-10A1), PDCA-1-FITC (clone 927), F4/80-APC-Cy7 (clone BM8), CD8α-APC (clone 53-6.7), CD45-AF700 (clone 560510), MHC class II-Biotin (clones 10-3.6 or M5/114.15.2; secondary reagent streptavidin-BV605; eBioscience). In experiments quantifying CD103^+^ APCs, this panel was modified by removing mAbs recognizing CD80, CD8α and PDCA-1, and adding mAbs recognizing CD103 (PE-coupled; clone 2E7) and CD40 (FITC-coupled; clone 3/23). Unless indicated otherwise all reagents were from BD Biosciences. 7-AAD was added to samples before analysis on a BD Fortessa™ cytometer, and data were analyzed using FlowJo™ (Tree Star, Ashland, OR). Statistical significance of differences was assessed using Mann-Whitney tests and Prism software (Graphpad, San Diego, U.S.A.).

### 2.4 Proteasome and IDE digestions

Recombinant human PI was provided by Eli Lilly. The protein resolved as a single band in reducing and non-reducing SDS-PAGE and as a single peak in reversed-phase chromatography on a µRPC C2/C18 column (GE Healthcare, Orsay, France). Recombinant rat IDE, purified from Sf9 insect cells by affinity chromatography followed by gel filtration (28), was a generous gift of L. Hersh, Univ. of Kentucky. 20S proteasome complexes, devoid of contamination by tripeptidyl-peptidase II, were purified by immunoaffinity chromatography from polyethylene-glycol 6000 precipitated cytosol fractions, using monoclonal antibody MCP21 (29) coupled to Sepharose 4B beads, as previously described (30). The lymphoblastoid human B cell lines 721 and 721.174 (31) were used as source for immuno- and constitutive proteasome complexes, respectively.

In a typical digestion, 40 µg PI was digested with 2 µg purified proteasome (molar ratio of ~1500:1) for 56 to 96 h. PI was either reduced and carboxymethylated as described (32), or reduced by incubation for 1 h at 37°C with 10 mM DTT before digestion, or directly used for digestions. In all cases, PI was first denatured using 0.1% sodium-dodecylsulfate (SDS) before addition of proteasome. Digestions were performed in 20 mM Hepes beuffer supplemented with 1 mM ethylene-bis(oxyethylenenitrilo)-tetraacetic acid, 5 mM MgCl_2_, 0.004% SDS and 1 mM DTT; digestions of oxidized PI were carried out in the absence of DTT. Digested material was fractionated by reversed-phase chromatography as described (32).

### 2.5 Mass spectrometry

Fractions were analyzed by matrix-associated laser desorption ionization/time of flight (MALDI-TOF) mass spectrometry (MS) with a Voyager STR spectrometer coupled with delayed extraction (Applied Biosystems, Warrington, U.K.). Mass accuracy was between 5 and 40 ppm. Mass signals resulting in ambiguous assignments were subjected to MS/MS analysis on a Micromass quadrupole time of flight instrument (Q-TOF; Waters, Elstree, U.K.). A Q-TOF instrument was also used to determine the molecular weight of untreated oxidized PI (9386.5 Da), PI reduced with 10 mM DTT and incubated for 20 h at 37°C in PBS (9388.0 Da) or Tris buffer (9388.8 Da) containing 1 mM DTT, and reduced and carboxymethylated PI (9745 Da). Expected values for fully oxidized, reduced, and reduced/carboxymethylated PI are 9388.1 Da, 9394.1 Da, and 9744 Da, respectively. PI digestions by IDE, fractionation and MS analysis were performed using the same buffer and the same molar substrate excess as in proteasome digestions, but without using reducing agents. One out of two digestions is shown.

### 2.6 Splenocyte transfers

Spleens were obtained from non-diabetic *Ide^+/+^* or *Ide^-/-^* mice, teased, filtered and subjected to red blood cell lysis by incubation in 5 ml of 150 mM NH_4_Cl, 1 mM KHCO_3_, and 0.1 mM EDTA for 5 min. B cells were depleted by staining with a biotinylated Ab against B220 (clone RA3-6B2; all Abs from Becton Dickinson, Le Pont-de-Claix, France) followed by incubation with paramagnetic streptavidin beads (Miltenyi Biotec, Paris, France). The remaining cells were stained with a biotinylated CD25 Ab (clone 7D4) and two fractions were obtained by paramagnetic bead sorting: the CD25^+^ fraction was used for transfers as “regulatory T cells”, while the CD25^-^ cell fraction was further purified by depleting CD62L^+^ cells again using a biotinylated Ab (clone MEL-14) and paramagnetic beads. The CD25^-^ CD62L^-^ fraction was used for transfer of “activated T cells”. The purity of the cell populations was tested by staining with Abs against CD4 (clone RM4-5; PercP-conjugated), CD25 (clone PC61; APC), and CD62L (clone MEL-14; PE). Six-week-old *Rag2^-/-^* NOD mice were injected in the retro-orbital vein with a single cell population or with a mixture of two cell populations: 5 x 10^5^ CD25^-^CD62L^-^ cells alone, 5 x 10^6^ splenocytes alone from a diabetic mouse as positive control, or co-transfer of 5 x 10^6^ splenocytes from a diabetic mouse with 5 x 10^5^ CD25^+^ cells. Glycosuria was monitored weekly after transfer.

### 2.7 Islet grafts

Hand-picked islets were transplanted under the left kidney capsule of mice at onset of hyperglycemia using a retroperitoneal approach. Briefly, under anesthesia, the left kidney was exposed, and a pouch was formed between the capsule and the kidney parenchyma of about 7×12 mm size. 500 islets were transferred into the kidney pouch. The blood glucose concentration was monitored daily after islet transplantation. Loss of graft function was defined as a blood glucose concentration > 300 mg/dL on two consecutive days.

### 2.8 MHC-I antigen presentation assays

Presentation of the insulin B_15-23_ epitope was examined using TCR-transgenic G9C8 CD8^+^ T cells. Freshly obtained splenocytes, or thawed enriched G9C8 cells, were resuspended in Click’s medium complemented with 10% FCS, 2 mM L-glutamine, 10mM HEPES and 50μM 2-mercaptoethanol, and restimulated by mixing at a ratio of 1:10 with irradiated NOD splenocytes pulsed with 10^-6^ M peptide B_15-23_ (LYLVCGERG; Schafer-N, Copenhagen, Danemark), with addition of TCGF supernatant of rat splenocytes on days 2 and 3. G9C8 cells were used for experiments on day 7 after 1 to 3 restimulations. To measure G9C8 stimulation, cells were stained at 4°C in PBS containing 2% FCS and 0.5% EDTA, 0.1% sodium azide after FcγRII/III blocking. Surface staining was performed with antibodies to CD45 (clone 30-F11), CD8 (clone 53-6.7) and IFN-γ (clone XMG1.2; all eBioscience). IFN-γ production was measured using intracellular cytokine staining (Biolegend) after a 6h incubation with islet cells in the presence of brefeldin A (5 μg/mL), or a 16h incubation with a protein transport inhibitor cocktail (eBioscience). Finally, cells were analyzed on a FACS Fortessa flow cytometer (BD Biosciences).

Generally, experiments were performed by mixing 5x10^4^ dissociated islet cells with 2x10^5^ G9C8 cells. To test proteasome inhibitors, islet cells were pre-cultured for 16h with 0.2 μM ONX-914 or 5 μM epoxomicin before incubation with G9C8 cells for 16h (direct presentation). To examine the role of ERAAP, *Ide^+/+^* mice were back-crossed twice to *Eraap^-/-^* mice (33), to obtain *Ide^+/+^Eraap^-/-^* mice. To assess the effect of ER stress, *Ide^+/+^* mice were injected *i.p.* with tunicamycin (2 mg/kg; Sigma) alone or together with rapamycin (5 mg/kg per day; Sigma), or with PBS, and G9C8 stimulation was measured after 48h. To test cross-presentation, BM-DCs were differentiated as described (34). Islets used as antigen donors were pre-cultured overnight to remove infiltrating lymphocytes (35) and then killed by freeze-thawing. Then 10^5^ BM-DCs per well were incubated overnight with 2 x 10^5^ killed islet cells before addition of 2x10^4^ G9C8 cells, an incubation for 12h and flow cytometric analysis as described above.

### 2.9 ^51^Cr release assay

Islets from 9-week-old female *Ide^+/+^* and *Ide^-/-^* mice were loaded with 200 mCi [^51^Cr] sodium chromate (Amersham Pharmacia Biotech, Piscataway, NJ) for 90 min, then plated in a U-bottomed 96 well plate at 10 islets/well. Target cells were incubated with G9C8 cells at an effector:target ratio of 20:1 and 10:1 in triplicate for 16h at 37°C. Medium alone or 2% Triton X-100 was added to a set of target cells to determine spontaneous and total cell lysis, respectively. The radioactivity of harvested supernatant was measured on a gamma counter (Perkin-Elmer). Specific ^51^Cr release was calculated as: percent lysis = (test cpm – spontaneous cpm)/(total cpm – spontaneous cpm) ×100.

### 2.10 Assessing stimulation of BDC2.5 CD4^+^ T cells

Islet antigens were prepared by subjecting hand-picked islets suspended in 0.5 x PBS to three cycles of freezing in liquid nitrogen followed by thawing at 37°C. Particulate islet fragments were sedimented by centrifugation, re-suspended in complete RPMI and added at different ratios to BM-DCs. To assess stimulation of BDC2.5 cells (36), red blood cell-depleted splenocytes from BDC2.5 transgenic mice (37) were stained with Abs to CD4 (clone RM4-5), CD25 (clone PC61) and CD62L (clone MEL-14; all from BD Biosciences), and naïve cells were sorted as CD4^+^CD25^-^CD62L^+^ cells using an Aria cell sorter (BD). Washed live cells were labeled using 5 μM carboxyfluorescein succinimidyl ester (CFSE) and added at 2.5 x 10^4^ per well in duplicates to BM-DCs plus islet debris or 1μM mimotope p31 (38) as positive control. After a 4-day incubation, proliferation was measured as percentage of lymphocytes having undergone at least 1 division, using a Fortessa™ instrument (BD).

### 2.11 Bone marrow transfers

To obtain bone marrow cells, the hind legs of TCR-transgenic G9C8 *Rag2^-/-^* or 8.3 *Rag2^-/-^* female or male mice were excised, skinned and shipped with attached muscles and ligaments in cold RPMI to INSERM U1151. Bone marrow was harvested by flushing of the long bones. The cell suspension was filtered through a 40 μm cell strainer, washed twice in PBS and either injected directly or frozen for later use. Eleven-week-old recipient mice were irradiated at 12 gray and injected with 5 x 10^6^ bone marrow cells in PBS via the retro-orbital vein. The efficacy of irradiation was controlled by staining white blood cells for CD8 and CD4 3 days after irradiation which showed <1% lymphocytes among blood cells and < 1% CD8^+^ and <5% CD4^+^ cells among lymphocytes, with no difference between *Ide^+/+^* and *Ide^-/-^* mice. Non-reconstituted control mice died between 6 and 12 days after irradiation.

### 2.12 Determination of diabetes incidence

Mice were checked thrice weekly for glycosuria using Diabur-Test 5000 strips (Roche). A positive result was verified by checking glycemia using an Accu-Chek. Mice with two consecutive readings ≥ 250 mg/dL were considered diabetic.

### 2.13 Insulitis scoring

Pancreata were fixed in 4% formalin for at least 2 hrs, embedded in paraffin overnight and mounted in a paraffin block. Four µm paraffin sections were mounted onto Superfrost™ slides coated with albumin, dried, deparaffinized and re-hydrated in 100%, 90%, 80% alcohol baths. Sections were stained in hematoxylin and eosin, each for 2 min, and mounted with EUKIT and a coverslip. For each pancreas, 30 islets were scored for insulitis (39).

### 2.14 Statistical tests

Significance of differences was evaluated using one or two-tailed Mann Whitney tests and Mantel-Cox tests for survival curves.

## 3 Results

### 3.1 Autoreactive T cell responses in IDE-deficient NOD mice

**Figure 1:**
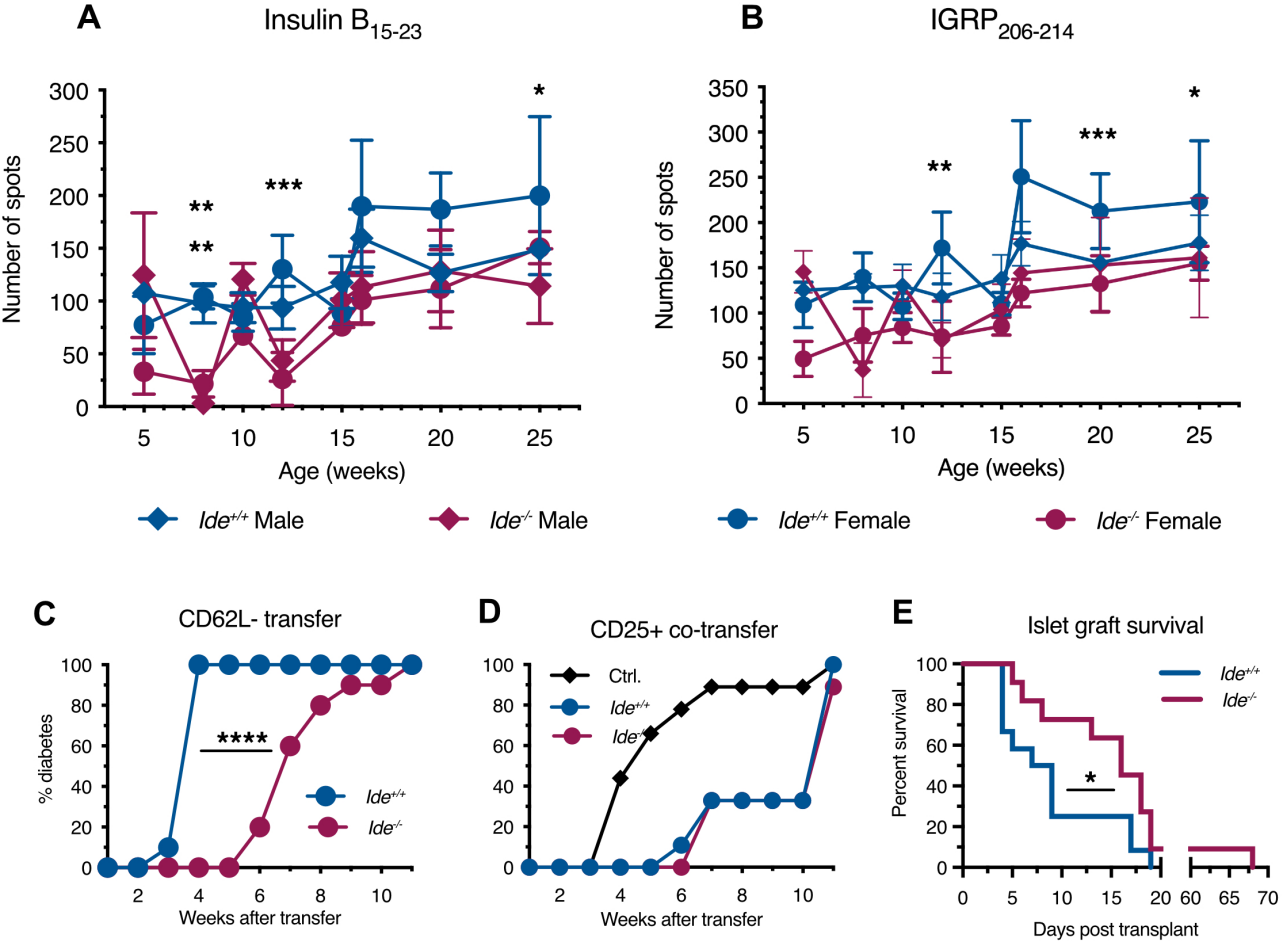
T cell responses against islet cell antigens in Ide^-/-^ NOD mice. **(A, B)** Recognition of immunodominant islet cell epitopes by peripheral blood lymphocytes from mice of different age (n=4 per group) was tested by IFN-γ ELISPOT assay. Lymphocytes were incubated with irradiated splenocytes and peptides for 24 hrs. **(A)** Recognition of insulin B_15-23_. **(B)** Recognition of IGRP_206-14_. Statistical significance was assessed by two-way Anova with Sidak’s multiple comparison test. Statistical significance was reached at 8, 12 and 25 weeks for female responses, and at 8 weeks only for male responses to B_15-23_. Responses to IGRP_206-214_ were significantly different only for female mice at 12, 20 and 25 weeks. *** p<0.001, ** p<0.01, * p<0.05 **(C)** Splenocytes from non-diabetic female mice were enriched for effector T cells by removing B220^+^ B cells and then CD62L^-^ naïve cells and CD25^+^ regulatory cells. The remaining cells were injected i.v. into 6-week-old *Rag^-/-^Ide^+/+^* and *Rag^-/-^Ide^-/-^* recipients, followed by weekly monitoring of diabetes incidence. N=9 or 10 per group. p<0.0001 in Mantel-Cox test. **(D)** Splenocyte fractions were prepared as in **(C)**, but here B220^-^CD25^-^CD62L- effectors from a diabetic mouse were transferred to *Rag^-/-^* recipients alone (Ctrl.) or together with a ten-fold lower number of B220^-^CD62L^-^CD25^+^ lymphocytes enriched for regulatory cells obtained from a non-diabetic *Ide^+/+^* or *Ide^-/-^* mouse. N=9 or 10 per group. **(E)** Recently diabetic mice received 500 *Ide^+/+^* or *Ide^-/-^* islets previously depleted of infiltrating lymphocytes by overnight incubation, followed by monitoring of glycemia until graft rejection. N=12 recipients for *Ide^+/+^* and 11 for *Ide^-/-^* islets. p=0.037 in Gehan-Breslow-Wilcoxon test.

To obtain initial evidence on a potential role of IDE in autoreactive T cell responses in the NOD model, we decided to examine the levels of CD8^+^ T cells recognizing beta cell antigens known to play a prominent role in T1D pathogenesis (40). As autoreactive islet-infiltrating and peripheral blood CD8^+^ T cells display similar specificity (41), we monitored IFN-γ production by peripheral blood lymphocytes in response to the immunodominant CD8^+^ T cell epitopes, insulin B_15-23_ and islet-specific glucose-6-phosphatase catalytic subunit-related protein (IGRP)_206-14_ (42). In both male and female mice, the number of responding T cells increased with age to reach a plateau at 16 weeks (**Figures 1A, B**). However, *Ide^-/-^* mice of both sexes had lower numbers of responding cells than *Ide* ^+/+^ mice at all ages. Therefore, *Ide* deficiency was associated with decreased autoreactive CD8^+^ T cell responses in peripheral blood.

T1D results from a shift of the equilibrium between auto-aggressive and regulatory lymphocytes towards the former. We performed adoptive transfer experiments of lymphocyte sub-populations to evaluate the presence and function of pathogenic and protective T cells in *Ide^-/-^* vs. *Ide^+/+^* mice. Transfer to *Rag^-/-^* NOD mice of activated splenocytes from *Ide^+/+^* non-diabetic donor mice that had been depleted of B cells, CD62L^+^ naïve and CD25^+^ “regulatory” T cells (i.e. that were enriched in effector T cells) (**Supplementary Figure S1**), resulted in diabetes in all recipients by 4 weeks (**Figure 1C**). In contrast, development of diabetes started 6 weeks post infusion of *Ide^-/-^* effector T cells and reached 100% only at 11 weeks. To examine regulatory T cell function, we performed adoptive co-transfer experiments, mixing activated splenocytes from an *Ide^+/+^* mouse with CD25^+^ splenocytes enriched in regulatory T cells from *Ide^+/+^* or *Ide^-/-^* mice. While transfer of activated splenocytes from *Ide^+/+^* mice alone resulted in diabetes onset at 4 weeks, reaching 90% by 7 weeks, co-transfer of “regulatory” splenocytes from both *Ide^+/+^* and *Ide^-/-^* delayed first appearance of diabetes to 6 or 7 weeks post-infusion, 90% diabetes being reached at 11 weeks (**Figure 1D**). These results suggested that *Ide^-/-^* mice had reduced numbers of pathogenic but equal numbers of “regulatory” T cells among splenocytes. Considering that protection from T1D by regulatory T cells in islet infiltrates might not necessarily be reflected in increased activity of splenic CD25^+^ T cells, we also examined the phenotype of islet-infiltrating CD4^+^ and CD8^+^ T cells from mice aged 9 and 20 weeks (**Supplementary Figure S2**). The frequency of activated (CD69^+^ or CD44^+^) CD8^+^ T cells did not differ between *Ide^+/+^* and *Ide^-/-^* mice. Similarly, *Ide^+/+^* and *Ide^-/-^* mouse infiltrates contained identical proportions of total, activated (CD69^+^) and regulatory (CD25^+^ or Foxp3^+^) CD4^+^ T cells at both ages. Thus, examination of islet infiltrate phenotypes also did not provide evidence for a role of increased frequency of regulatory T cells in protection of *Ide^-/-^* mice from T1D.

To test whether *Ide^-/-^* islets might display increased resistance to T1D induction, we grafted infiltrate-depleted *Ide^+/+^* and *Ide^-/-^* islets into recently diabetic *Ide^+/+^* NOD mice and monitored the time to autoimmune graft destruction/rejection. While most *Ide^+/+^* grafts were destroyed by day 9 after transplantation, the same rate of graft destruction was reached only on day 17 post-transplant for *Ide^-/-^* islets, and 1 graft survived for almost 10 weeks (**Figure 1E**). Therefore, IDE deficiency not only resulted in reduced numbers of autoreactive CD8^+^ T cells but also in reduced rejection of *Ide^-/-^* islets by wild-type T cells, potentially mediated by attenuated recognition of autoantigens.

### 3.2 PI digestion by the proteasome and IDE

Considering that IDE deficiency attenuated pathogenic T cell activity and islet rejection, we speculated that altered presentation of insulin peptides to cytotoxic T cells by beta cells might contribute to this finding. To address this, we first sought to determine the digestion profiles of PI by purified proteasome complexes and IDE *in vitro*.

**Figure 2:**
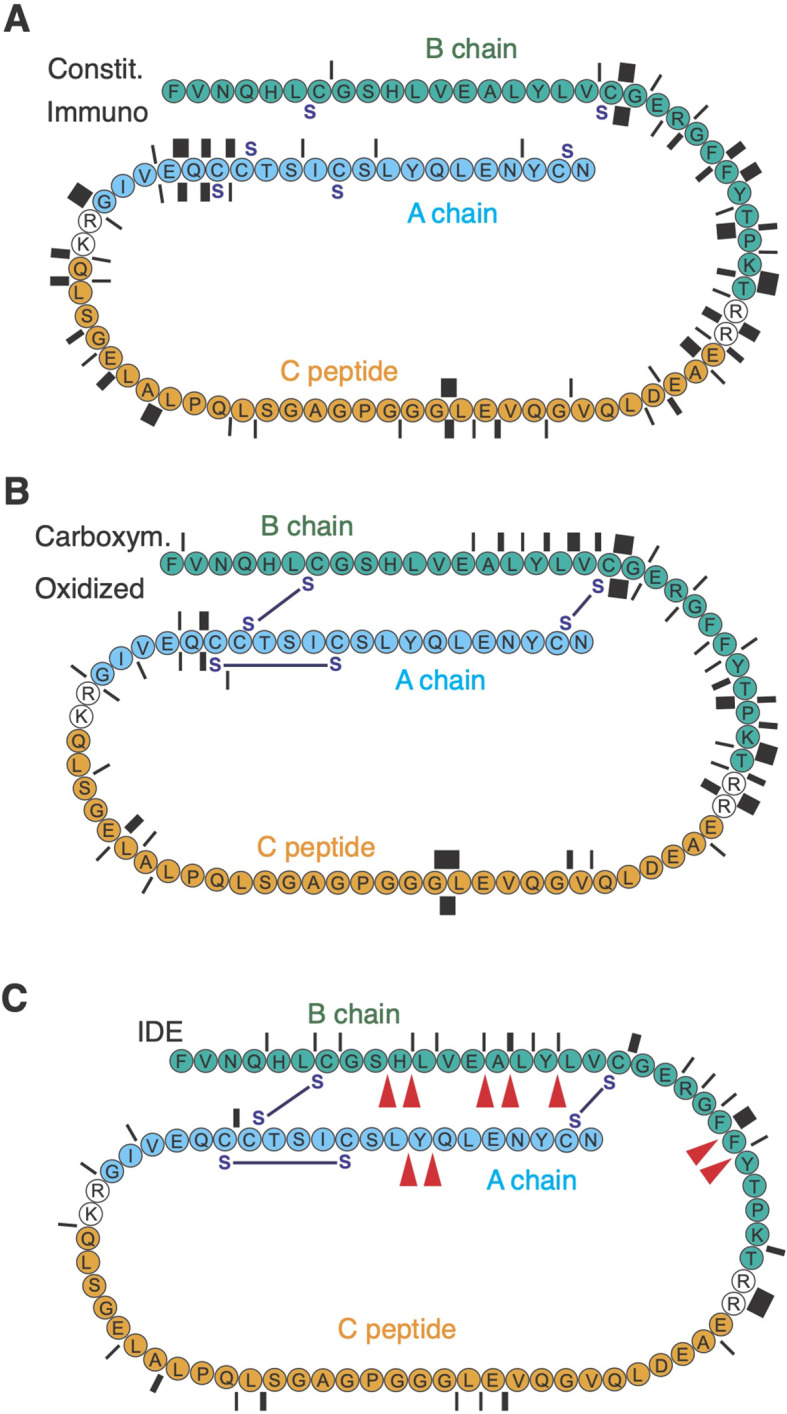
PI degradation by 20S proteasome complexes and IDE. Protease cleavage products were identified by mass spectrometry. In **(A)** PI (represented N- to C-terminal), previously reduced with 10 mM DTT, was digested in the presence of 1 mM DTT with immunoaffinity-purified immuno- or constitutive proteasome for 56h. Cleavage sites are indicated by bars whose thickness corresponds to the number of cleavages (between 1 and 8). In **(B)** reduced and carboxymethylated PI, or PI never exposed to DTT (oxidized), were digested for 56h with 20S proteasome. **(C)** shows the number of cleavages (between 1 and 8) obtained in digestions of oxidized PI for 56 h with IDE. Red arrowheads indicate the published main cleavage sites of IDE.

PI consists of the insulin core formed by the A and B chains linked by three disulfide bridges, and the structurally disordered connecting peptide removed during insulin maturation (**Figure 2**). We digested recombinant human oxidized PI with human 20S core proteasome complexes. In initial digestions, PI was reduced with 10 mM dithiotreitol (DTT), and then digested in the presence of 1 mM DTT. Mass spectrometric (MS) mapping of cleavage site usage showed clustering of cleavages around the junctions of the C peptide with the A and B chains, whether immuno- or constitutive proteasome complexes were used (**Figure 2A**; **Supplementary Figure S3**). Surprisingly, cleavages were rare in the proximal B chain and distal A chain although the isolated oxidized B chain has been shown to be an efficient proteasome substrate (10). Reasoning that this absence might reflect re-formation of disulfide bridges preventing proteasome digestion of the enclosed moieties, we determined the molecular weight of DTT-treated PI and found it to differ from fully oxidized PI by 2 Da. This was consistent with two of the three disulfide bridges having re-formed. Digestion of fully oxidized PI, never exposed to DTT, indeed phenocopied the results obtained with DTT-treated PI (**Figure 2B**). Conversely, additional cleavages between B7 and B19 were observed when carboxymethylated PI was digested to prevent re-oxidation of PI (**Figure 2B** “Carboxym.”). However, although the proteasome could cleave at the N-terminus of insulin B_15-23_ (LYLVCGERG), no cleavage at the C-terminus was observed. Thus, the proteasome is largely unable to digest oxidized PI which can form even in a reducing environment. Consequently, akin to some other larger proteins (43), newly synthesized PI might exit the ER to the cytosol in a fully folded, oxidized form resistant to proteasome digestion.

**Figure 3:**
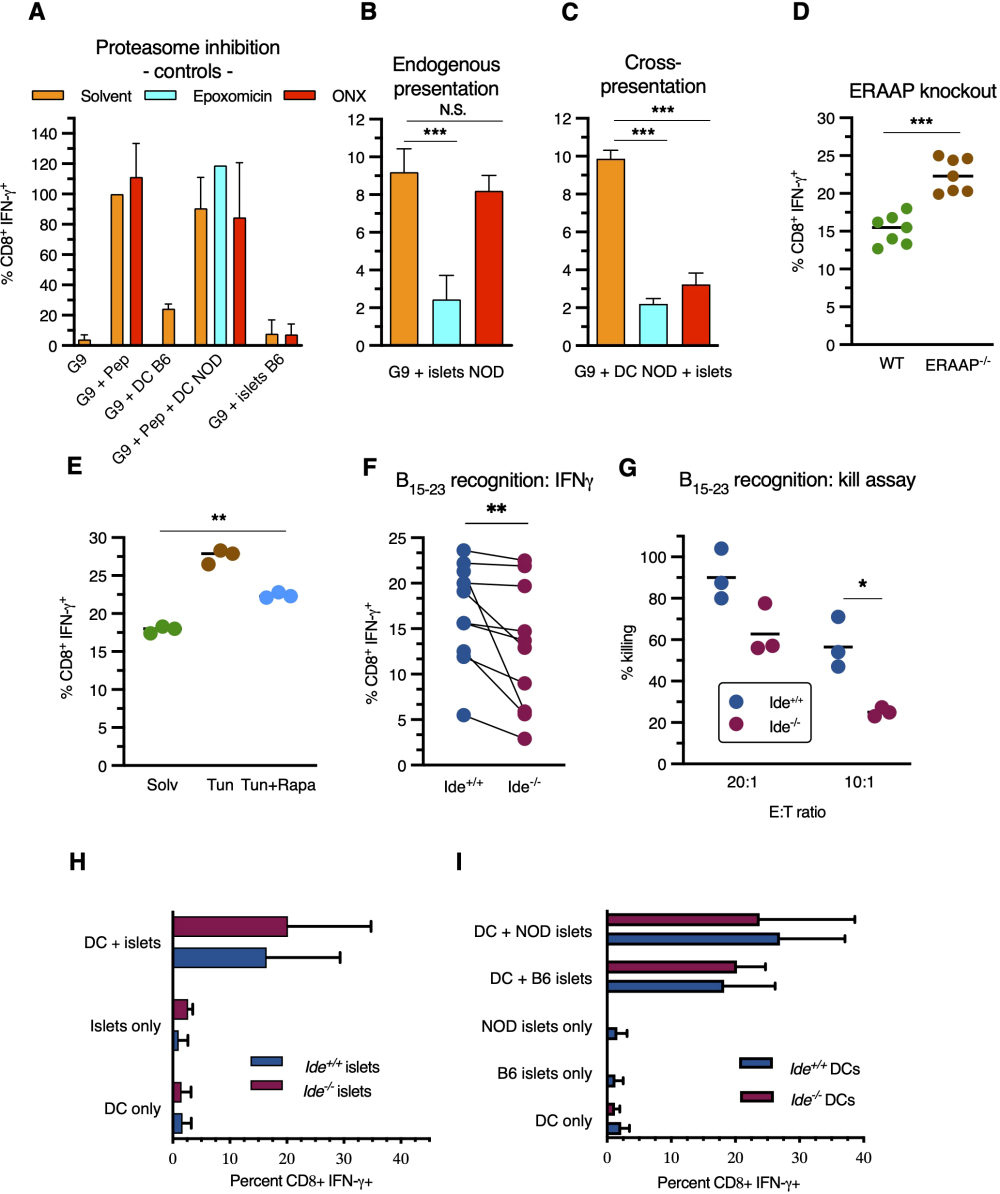
Antigen processing pathways of insulin B_15-23._ **(A–F)** G9C8 T cell stimulation was examined using intracellular IFN-γ staining for flow cytometric analysis. **(A)** G9C8 cells were incubated (left to right) alone (“G9”), with 10^-6^M peptide insulin B_15-23_, with C57BL/6 BM-DCs, with NOD BM-DCs plus 10^-6^M peptide insulin B_15-23,_ and with live C57BL/6 islets, in the presence of epoxomicin or ONX-914. Results are expressed as percent of the positive control (G9C8 cells with peptide) set at 100% in **(A)**, and as (unprocessed) percent CD8^+^IFN-γ^+^ in other panels. G9C8 stimulation by peptide in the absence of APCs added is due to carry-over of splenocytes used for activation/restimulation of G9C8 cells prior to the experiments. **(B, C)** G9C8 cells were incubated **(B)** with live NOD islets, or **(C)** with killed C57BL/6 islets plus NOD BM-DCs, in the presence of proteasome inhibitors. **(D)** NOD mice were crossed with *Eraap-/-* C57BL/6 mice to obtain *Eraap^-/-^* H-2K^d^ F2 mice and stimulation of G9C8 cells by live islets was tested. **(E)** NOD mice were treated with tunicamycin ± rapamycin followed by isolation of live islets for stimulation of G9C8 cells. **(F)** IFN-γ production by G9C8 cells stimulated by *Ide^+/+^* and *Ide^-/-^* live islets was compared. p=0.002 in Wilcoxon matched-pairs rank test. **(G)** G9C8 cells were added to *Ide^+/+^* and *Ide^-/-^* islets obtained from 9-week-old mice at an effector to target ratio of 20:1 and 10:1 and killing of ^51^Cr-labelled islet cells was measured. Data in panels **(B–D, F, G)** were evaluated by Mann-Whitney test, and data in panel **(E)** by Kruskal-Wallis one-way Anova. **(H, I)** Cross-presentation of insulin: hand-picked islets were pre-incubated overnight to remove infiltrating lymphocytes, dissociated to single cells and added to BM-DCs overnight before addition of G9C8 cells for 12 hrs, G9C8 staining for intracellular IFN-γ and flow cytometric analysis. **(H)** Freeze-thaw killed *Ide^+/+^* or *Ide^-/-^* C57/BL6 islets were added to *Ide^+/+^* NOD BM-DCs. “DC only” indicates stimulation of G9C8 cells in the presence of BM-DCs, and “islets only” stimulation in the presence of islets and absence of DCs. N=7. **(I)** Like in **(H)**, but *Ide^+/+^* NOD or C57BL/6 islets were added to *Ide^+/+^* or *Ide^-/-^* BM-DCs. Graphs show means ± SDEV. N=4. *, p<0.05; **, p<0.01; ***, p<0.001; N.S., not significant.

IDE has been shown to cleave complete oxidized insulin efficiently, using a number of consensus cleavage sites in the B and A chains reported by multiple authors (44–48). We confirmed these sites showing that IDE produces very similar cleavages in fully oxidized PI, including a frequent cut after Alanine14 that produces the N-terminal of the epitope B_15-23_ (**Figure 3C**). The latter cleavage occurs early during insulin degradation by IDE (44) and may therefore initiate production of insulin B_15-23_. Note, that IDE also could cleave at the C-terminus of B_15-23_ though with low efficacy (**Figure 3C**).

### 3.3 Antigen presentation pathways involved in insulin B_15-23_ presentation to CD8^+^ T cells

To determine how these results of *in vitro* digestions correlated with cellular antigen processing, next we performed cellular antigen presentation assays to determine the poorly defined pathways involved in presentation of the highly immunodominant epitope insulin B_15-23_ to CD8^+^ T cells. This was done using cognate clonal CD8^+^ T cells obtained from TCR-transgenic G9C8 mice (49). Antigen recognition by G9C8 cells was detected by intracellular flow cytometric staining for IFN-γ of CD45^+^CD8^+^ TCR-Vβ6^+^ cells after incubation with beta cells or professional APCs, supplemented with synthetic peptide insulin B_15-23_ or not, which activated up to 40% of G9C8 cells (**Supplementary Figure S4**).

G9C8 cells were activated by synthetic peptide in the presence or absence of NOD dendritic cells (DCs) while C57BL/6 islets or DCs had no effect, as expected (**Figure 3A**). Addition of epoxomicin, a general proteasome inhibitor, or ONX-0914, a specific immunoproteasome inhibitor (50), had no effect on stimulation by synthetic peptide. Incubation with NOD islets stimulated IFN-γ expression, indicating that NOD islet (i.e. presumably beta) cells can present H-2K^d^/insulin B_15-23_ complexes to G9C8 cells (**Figure 3B**). This was inhibited by epoxomicin but not ONX-0914. In contrast, cross-presentation of killed islets by NOD DCs was inhibited by both inhibitors (**Figure 3C**). Thus, the insulin B_15-23_ epitope can be produced by both proteasome types. However, although mouse beta cells have been reported to express the β5i proteasome subunit targeted by ONX-0914 (51), only DCs express enough immunoproteasome to contribute to presentation. To determine whether the insulin epitope undergoes aminopeptidase trimming in the endoplasmic reticulum (ER), we crossed NOD mice to C57BL/6 mice lacking the ER aminopeptidase associated with antigen processing (ERAAP). Islets from *Eraap* ^-/-^ NOD mice displayed increased G9C8 stimulation, suggesting partial epitope destruction by ERAAP (**Figure 3D**). We also tested the effect of strong ER stress on insulin peptide presentation by beta cells. Tunicamycin, a drug inducing accumulation of unfolded proteins in the ER through inhibition of N-glycosylation, upregulated presentation, an effect abolished when protein production was inhibited (and thereby proteotoxic stress reduced) by the mTORC1 inhibitor rapamycin (**Figure 3E**). Thus, in beta cells, the presentation of the immunodominant insulin CD8^+^ T cell epitope implicates the constitutive proteasome and is downregulated by ERAAP but increased by beta cell stress.

Next, we asked whether IDE deficiency affected presentation. Although IFN-γ secretion by G9C8 cells exposed to islets varied between experiments, *Ide^-/-^* islets stimulated G9C8 cells less efficiently than *Ide^+/+^* islets (**Figure 3F**). Moreover, *Ide^-/-^* islets were significantly less susceptible to killing by G9C8 cells (**Figure 3G**). G9C8 cells incubated with *Ide^-/-^* islets for 8 or 16h expressed equivalent levels of programmed death-1 (PD-1), indoleamine dioxygenase (IDO) and CD122, indicating that resistance was not due to expression of one of these inhibitory molecules (**Supplementary Figure S5**). Collectively and consistent with the results of *in vitro* digestions, these results suggest that insulin or PI undergo cytosolic processing by both the proteasome and IDE for presentation by MHC class I molecules in beta cells.

Finally, we tested potential effects of IDE deficiency on activation of G9C8 cells by cross-presenting DCs, which play a key role in triggering islet inflammation (52). DC presentation of titrated synthetic peptide amounts was not affected by IDE expression (**Supplementary Figure S6A, B**). Moreover, *Ide^-/-^* islets from mice aged 4 to 12 weeks expressed similar levels of H-2K^d^ molecules as *Ide^+/+^* islets (**Supplementary Figure S6C**). Neither IDE expression by NOD islets as antigen donors for cross-presentation by NOD DCs (**Figure 3H**), nor IDE expression by NOD DCs cross-presenting *Ide^+/+^* islets (**Figure 3I**). influenced stimulation of G9C8 cells In summary, while IDE expression affected endogenous presentation of insulin B_15-23_ by beta cells, its deficiency in beta cells as antigen donors for professional APCs, or in these professional APCs after beta cell uptake, did not affect cross-presentation of the epitope.

We also considered the possibility that IDE might be implicated in the production of hybrid peptide epitopes generated by transpeptidation during cleavage of beta cell autoantigens. The CD4^+^ T cell clone BDC2.5 and TCR-transgenic CD4^+^ T cells expressing its receptor (37) recognize a hybrid formed by fragments of the chromogranin A and proinsulin autoantigens (53). We incubated bone marrow-derived NOD DCs with T cells from BDC2.5 TCR-transgenic mice and assessed proliferation after 5 days by flow cytometry. Incubation with DCs and islets induced dose-dependent T cell proliferation, however without any effect of *Ide* expression by islet cells (**Supplementary Figure S7**). Thus, IDE is unlikely to play a role in production of hybrid epitopes, a conclusion consistent with reports of a key role of cathepsin D in hybrid peptide production (54) and the absence of IDE from lysosomes.

### 3.4 IDE deficiency protects against diabetes transfer by insulin specific CD8^+^ T cells

**Figure 4:**
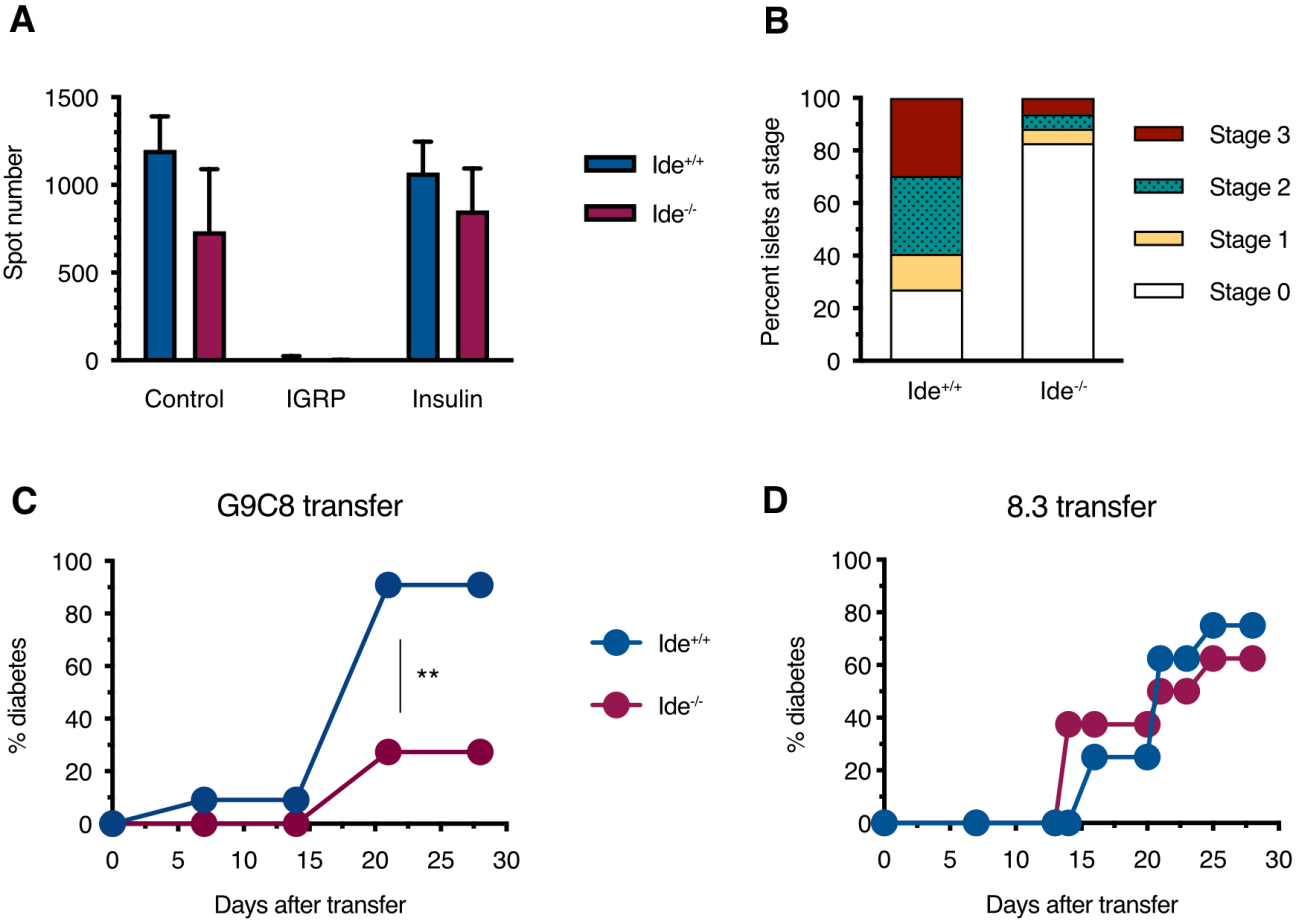
Diabetes induction in chimeras harboring monoclonal CD8^+^ T cell populations. **(A)** Sub-lethally irradiated female *Ide^+/+^* and *Ide^-/-^* NOD recipients were injected with bone marrow cells from G9C8 mice. Twenty-three days after receiving G9C8 bone marrow, peripheral blood lymphocytes from recipient mice were analyzed for IFN-γ secretion in response to IGRP_206-14_ and insulin B_15-23_ by ELISPOT. Control cells were stimulated with CD3 antibodies. N=8 per group. **(B)** Islets from the same bone marrow-grafted *Ide^+/+^* and *Ide^-/-^* mice were scored for infiltration. **(C)** Chimeras reconstituted with G9C8 bone marrow were monitored for diabetes. N=11 per group, **, p=0.0014 by Mantel-Cox test. **(D)** Experiment analogous to **(C)** but monitoring diabetes in recipients of bone marrow from 8.3 mice expressing a TCR recognizing the IGRP_206-14_ epitope. N=8 per group.

Considering the relative resistance of *Ide^-/-^* islets to killing/destruction by G9C8 cells *in vitro*, we wondered whether this was paralleled by protection against diabetes transfer by these diabetogenic T cells. To assess this, we reconstituted sublethally irradiated *Ide^+/+^* and *Ide^-/-^* mice with bone marrow from transgenic mice expressing CD8^+^ T cell receptors recognizing the immunodominant insulin B_15-23_ and IGRP_206-214_ epitopes. Twenty-three days after grafting, peripheral blood lymphocytes from *Ide^+/+^* or *Ide^-/-^* mice having received G9C8 bone marrow exclusively recognized the G9C8 cognate epitope (insulin B_15-23_) and not IGRP_206-14_, confirming efficient replacement of hematopoietic cells by the graft (**Figure 4A**). However, while 91% of *Ide^+/+^* recipients developed diabetes by 20 days after grafting, only 27% of *Ide^-/-^* recipients did so (**Figure 4C**). The lower T1D incidence was reflected in the extent of insulitis, with 83% of *Ide^-/-^* islets showing no infiltrate vs. 27% for *Ide^+/+^* islets (**Figure 4B**). In contrast, when we grafted mice with bone marrow from 8.3 mice recognizing IGRP_206-14_ (55), diabetes incidence was undistinguishable, with 63% of *Ide^-/-^* vs. 75% of *Ide^+/+^* mice being diabetic 25 days after grafting (**Figure 4D**). Therefore, IDE deficiency protects from T1D caused by CD8^+^ T cells recognizing an insulin peptide but not by T cells recognizing an IGRP peptide.

## 4 Discussion

In this study we set out to examine the role of IDE in the autoimmune diabetes model of the NOD mouse*. Ide^-/-^* mice had previously been recognized to display some features of T2D such as perturbed glucose tolerance and hyperinsulinemia, but a potential role in T1D had not been studied. We report that IDE deficiency reduces presentation of the immunodominant epitope insulin B_15-23_ and beta cell killing by cytotoxic T cells and protects from disease upon adoptive transfer of CD8+ T cells recognizing this epitope. Thus, IDE regulates processing of the key T1D autoantigen insulin in beta cells and thereby diabetes pathogenesis.

The processing of insulin for presentation by MHC class I molecules is incompletely understood (8), and processing of the immunodominant epitope B_15-23_ has not been studied previously. Importantly, the epitope B_15-23_ can be eluted from MHC molecules of islet cells (56), underlining the importance of analyzing its processing. We established that B_15-23_ presentation by freshly isolated beta cells requires the proteasome but is not affected by the immunoproteasome. Thus, although mRNA of the inducible beta5 subunit targeted by ONX-0914 has been detected in primary human and murine beta cells (51), its activity has no apparent impact on epitope processing in murine islets. Our failure to detect such an effect is not due to an inability of the immunoproteasome to produce the B_15-23_ epitope, as its cross-presentation by bone marrow-derived DCs (BM-DCs) is efficiently inhibited by ONX-0914. However, our results are consistent with observations by Hoelen and colleagues who studied PI degradation in PI-transfected K562 cells and found that PI processing depends on the proteasome and an ERAD (endoplasmic reticulum associated degradation) pathway implicating Derlin-2 (11). We also find evidence for destructive B_15-23_ trimming by ERAAP, and for upregulation of presentation by proteotoxic stress. The latter finding is reminiscent of observations made for a human insulin epitope encoded by an alternative open reading frame (57) and suggests that accumulating ER stress during T1D pathogenesis will increase the antigenicity of beta cells for key CD8^+^ T cells.

Our key finding is the role of IDE in endogenous presentation of insulin B_15-23_. Although IDE can interact with the proteasome with a regulatory effect (22, 23), and mediate antigen processing of a single tumor epitope (58), IDE has no broader role in MHC-I antigen processing (59). However, IDE has very high affinity for insulin and can also process intact PI (albeit less efficiently) (60, 61), consistent with our data, so that a role in cytosolic insulin and/or PI processing is plausible. IDE may exert its effect through interaction, or in concert with the proteasome or in a parallel ERAD pathway as has been shown for amyloid beta (62).

Considering published data and our observations, we propose the following hypothetical model for production of the CD8^+^ T cell epitope B_15-23_ in beta cells. PI failing to mature in the ER is retro-translocated through an ERAD-L pathway (63) in at least partly oxidized form. In the cytosol, both the proteasome and IDE can degrade PI, with preferential and initial cleavage of oxidized forms after Ala14 by IDE which may facilitate denaturation and downstream cleavage by the proteasome. Both enzymes display limited efficacy on their own due to relative proteasome resistance of oxidized PI, and to lower IDE affinity for oxidized PI relative to insulin (60). Processing may be assisted by a cytosolic or ER-luminal carboxypeptidase as only IDE cleaves after Gly25 albeit with low efficacy [**Figure 2** and (44)].

Besides altered B_15-23_ presentation as the key mechanistic finding/discovery in this study, *Ide^-/-^* NOD mice displayed evidence of a more broadly altered autoreactive immune response. This included a reduced number or efficacy of diabetogenic splenocytes, a lower number of circulating CD8^+^ T cells reacting to both insulin and IGRP, increased resistance of islet grafts to autoimmune destruction, and significantly lower islet infiltration both in spontaneous diabetes development and in chimeras reconstituted with G9C8 bone marrow (shown in reference 22). All these findings likely are consequences of diminished T cell responses to insulin. Insulin is a very early and possibly the earliest target of beta cell reactive CD8^+^ T cells in the NOD model (64), and T cell responses to key insulin epitopes condition initiation of autoimmunity against beta cells, with responses to epitopes of other autoantigens including IGRP occurring later (6, 65).

We did not find evidence for alternative explanations for attenuated autoimmunity in *Ide^-/-^* mice, as the efficacy and/or number of regulatory splenocytes and the phenotype of islet-infiltrating T cells were not significantly different compared to *Ide^+/+^* mice. Moreover, IDE expression in islets cells did not affect cross-presentation of B_15-23_ nor did IDE expression in cross-presenting BM-DCs. Conversely, the unaltered diabetogenic effect of IDE deficiency in mice reconstituted with 8.3 bone marrow confirmed the selective impact of this enzyme on insulin-specific CD8^+^ T cell responses, leading to reduced autoimmunity in *Ide^-/-^* mice.

In summary, we show that IDE promotes pathogenic cellular autoimmune responses to insulin through production of a key CD8^+^ T cell epitope in beta cells. Consequently, IDE deletion reduces islet inflammation and diabetogenic T cell responses, which are then likely to protect from spontaneous disease. Interestingly, IDE deficiency triggers at the same time a low-level unfolded protein response in islets which in turn induces significant beta cell proliferation and dramatically upregulates Reg2 (22), a protein known to promote beta cell regeneration and inhibit inflammation (66). Thus, IDE deficiency may protect from diabetes both by attenuating beta cell recognition by autoreactive T cells and by triggering an intrinsic regenerative beta cell defense.

### 4.1 Limitations of this study

Although the molecular weight of fully oxidized and of reduced and carboxymethylated PI corresponded to the expected values, the weight of DTT-treated PI did not, a finding possibly related to low stability of reduced PI resulting for example in disulfide bridge mispairing (67, 68). Moreover, as we did not monitor stability of the different PI forms over time in the absence of proteases, background degradation events contributing to production of PI fragments cannot entirely be ruled out. While our data pinpoint a role of reduced insulin presentation to cytotoxic T cells, the precise impact of this reduction in protection from spontaneous diabetes is difficult to decipher as upregulation of intrinsic beta cell defenses (proliferation, REG2 expression) may also contribute to protection.

## Data Availability

All data are available in the main text or the **Supplementary Material**. Primary data are available from the communicating author upon request. Further inquiries can be directed to the corresponding author.
