# Supplementary material for: Insulin-degrading enzyme regulates insulin-directed cellular autoimmunity in murine type 1 diabetes
Source: Front Immunol. 2024 Nov 12;15:1474453. doi: 10.3389/fimmu.2024.1474453 (PMC11588737; doi:10.3389/fimmu.2024.1474453)
Supplement: Supplementary file 1 [file DataSheet1.pdf]

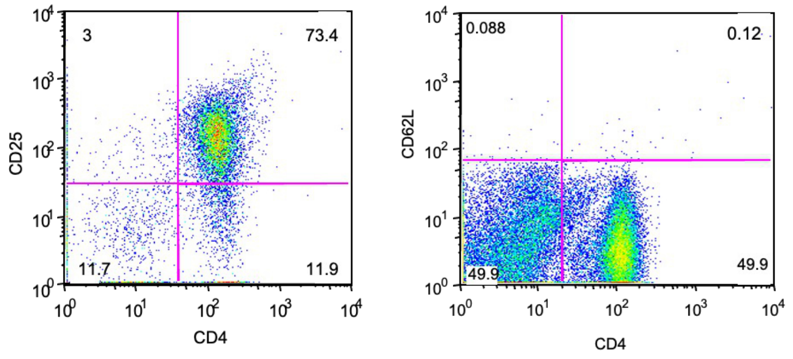

Figure S1. **Cell populations used for transfer experiments (**Fig. 1C,D**)**. Splenocytes from non-diabetic or diabetic *Ide*$^{+/+}$ or *Ide*$^{-/-}$ mice were subjected to sequential depletion of B220^{+} B lymphocytes followed by positive magnetic sorting of CD4^{+}CD25^{+} of a fraction enriched in regulatory T cells (left hand panel). The remaining fraction was further depleted of CD62L^{+} cells as source of "diabetogenic" T cells (right hand panel).

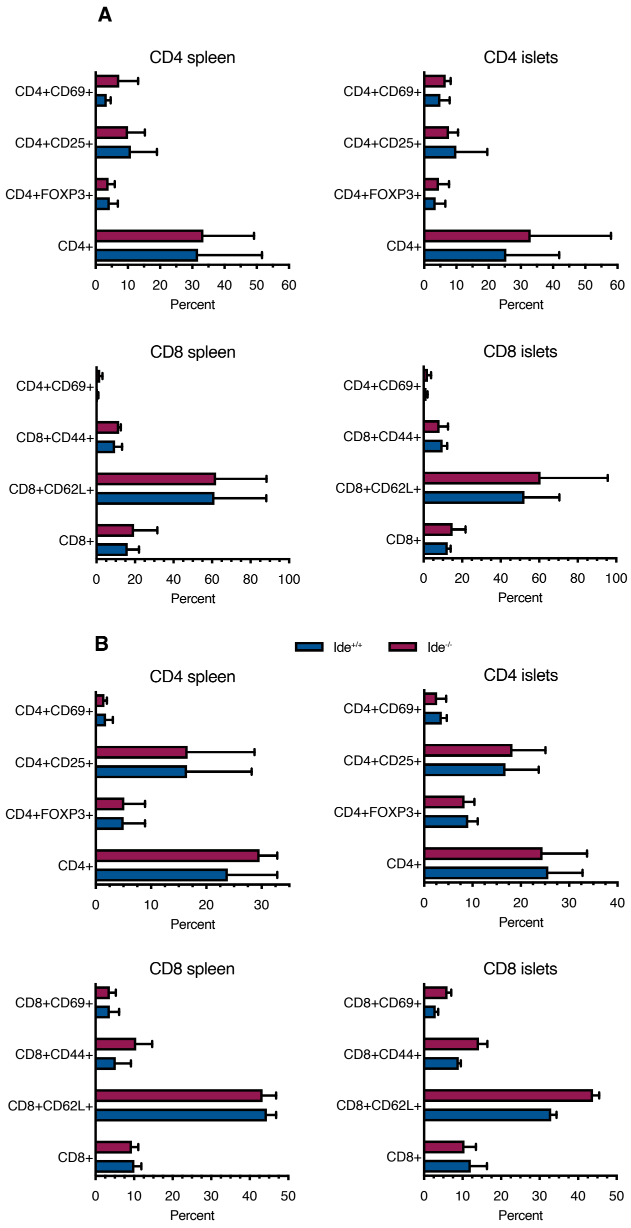

Figure S2. Phenotypes of splenocytes and islet-infiltrating T lymphocytes

Hand-picked islets and splenocytes were obtained from male *Ide*$^{+/+}$ and *Ide*$^{-/-}$ mice, dissociated to single cells, and lymphocytes were phenotyped by flow cytometry. The numbers indicate percentages among lymphocytes as identified using forward and side scatter. One of two experiments is shown. **(A)** Phenotype of islet-infiltrating T lymphocytes from mice aged 9 weeks. **(B)** Phenotype of islet-infiltrating T lymphocytes from mice aged 20 weeks. N=8 for splenocytes; N=2 pooled cells from 4 mice for islet infiltrating lymphocytes for panels A and B.

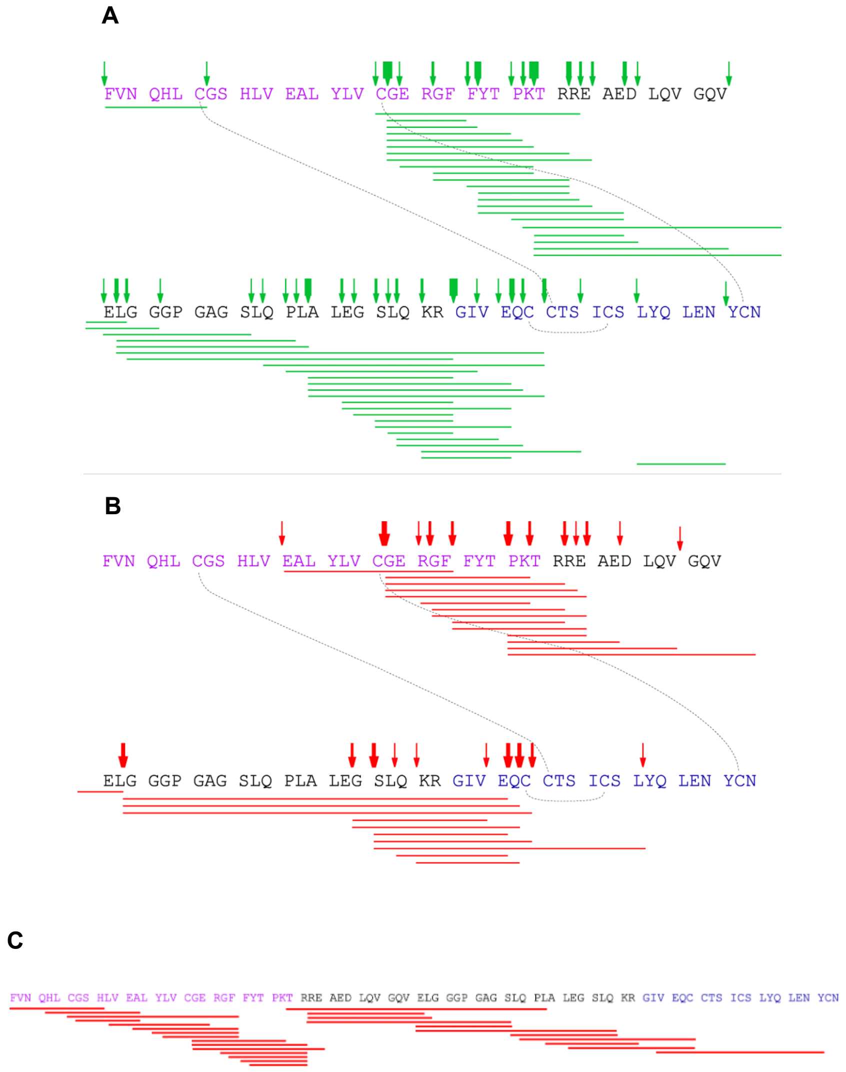

Figure S3. Proinsulin fragments as detected by mass spectrometry.

Lines show the position of individual proinsulin fragments produced by digestion of proinsulin with constitutive proteasome (A), immunoproteasome (B) or IDE (C).

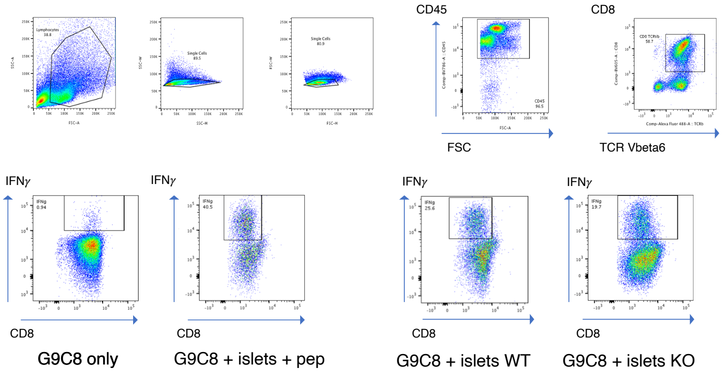

Figure S4. Gating for detection of G9C8 cells secreting IFN-γ

Representative flow cytometry dot plots showing detection of stimulated G9C8 cells in cocultures with islet cells as single CD45$^+$CD8$^+$TCR-Vβ6$^+$ cells staining for IFN-γ, as detected by intracellular cytokine staining.

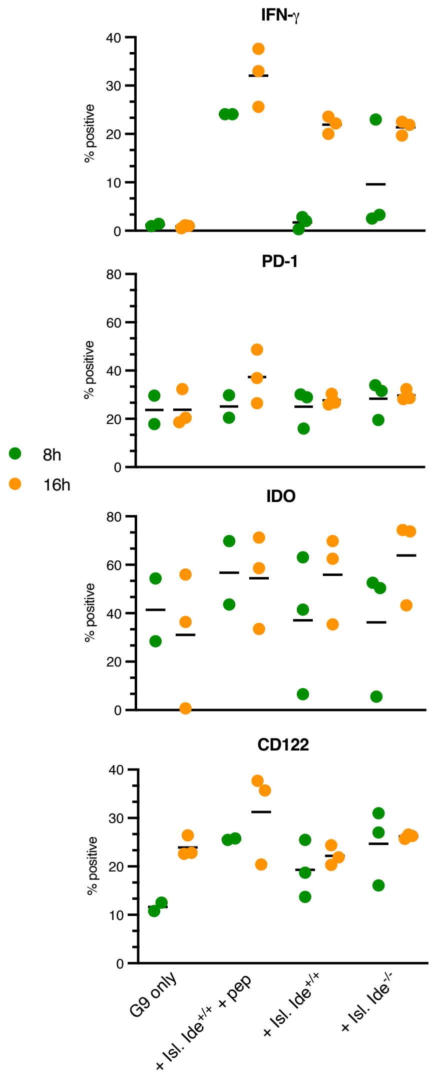

Figure S5. Expression of regulatory molecules by activated G9C8 cells

G9C8 cells (2 x 10^5) stimulated with peptide-pulsed splenocytes 7 days before were incubated alone, or with dissociated *Ide*$^{+/+}$ islets pulsed or not with 10^{-6} M peptide insulin $B_{15\text{-}23}$, or with dissociated *Ide*$^{-/-}$ islets. After 8h and 16h, cells were stained with antibodies to CD45, CD8, TCR Vβ6 as well as to PD1, CD122, and, after permeabilization, to IFN-γ and indoleamine 2,3-dioxygenase, and analyzed by flow cytometry. Each dot corresponds to islet cells from one mouse.

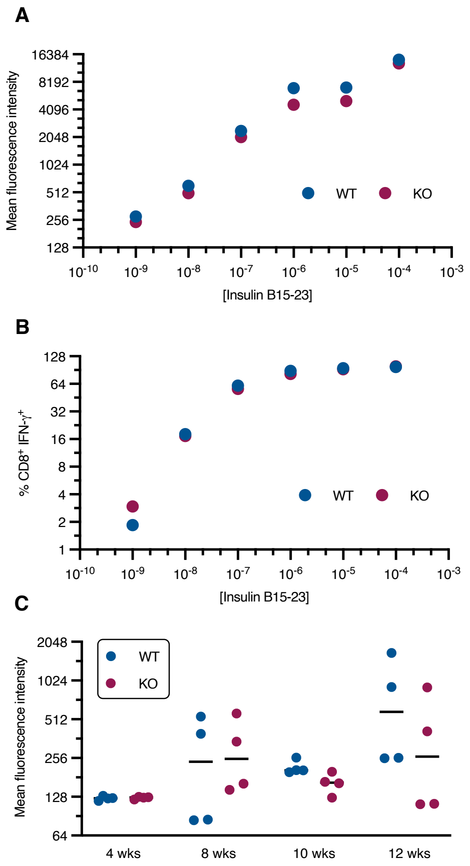

Figure S6. Synthetic peptide presentation and MHC-I expression by islet cells.

(**A, B**) G9C8 cells were added to BM-DCs from *Ide*$^{+/+}$ and *Ide*$^{-/-}$ mice pulsed with graded amounts of synthetic peptide insulin $B_{15\text{-}23}$, incubated for 16 hours and subjected to intracellular cytokine staining for IFN-γ, and analyzed by flow cytometry for mean fluorescence intensity (**A**) and percentage of IFN-γ^{+} cells (**B**). (**C**) Handpicked islets were prepared from female NOD mice aged 4 to 12 weeks, processed to single cell solutions and stained with antibodies to CD45 and H-2K^{d}. The histogram shows mean ±SDEV of the mean fluorescence intensity for H-2K^{d} among CD45 negative cells. N=4 per age and genotype group.

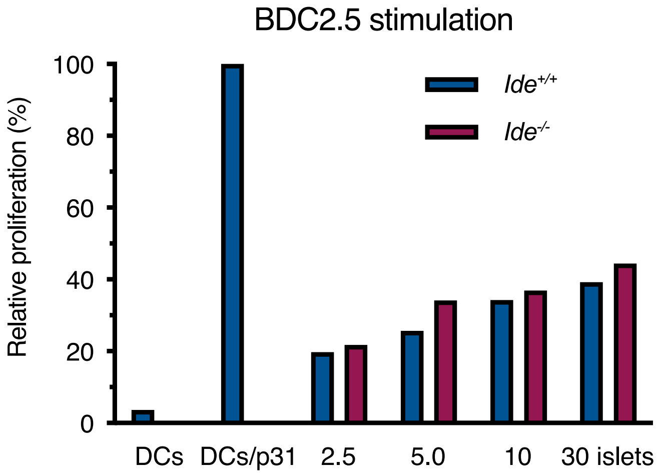

Figure S7. No IDE effect on generation of a hybrid CD4+ T cell epitope.

Ide$^{+/+}$ and *Ide*$^{-/-}$ C57BL/6 islets disrupted by repeated freeze-thawing were added to *Ide*$^{+/+}$ NOD BM-DCs and incubated for 5 days with CFSE-labeled, sorted CD4^{+}CD62L^{+}CD25^{-} naïve T cells from BDC2.5 TCR-transgenic. Then proliferation was assessed by flow cytometry. Data from 2 experiments performed in duplicate were normalized by expressing as percent of proliferation induced by DCs pulsed with the p31 synthetic cognate epitope.
